# Ca^2+^ microdomain-based excitation-transcription coupling in cardiac myocytes and vascular smooth muscle cells

**DOI:** 10.1186/s41232-025-00384-3

**Published:** 2025-06-23

**Authors:** Tsukasa Koide, Wayne R. Giles, Rubii Kondo, Yuji Imaizumi, Hisao Yamamura, Yoshiaki Suzuki

**Affiliations:** 1 Department of Molecular and Cellular Pharmacology, Graduate School of Pharmaceutical Sciences, Nagoya City University, Nagoya, Japan; 2 Department of Physiology and Pharmacology, Cumming School of Medicine, University of Calgary, Calgary, AB Canada

**Keywords:** Ca^2+^ signaling, Ca^2+^ microdomain, Excitation-transcription coupling, Cardiac myocyte, Vascular smooth muscle cell

## Abstract

Ca^2+^ signals play a crucial role in maintaining cardiovascular homeostasis, including regulation of the heartbeat, blood pressure, and adaptation to changes in the external environment. Conversely, abnormal Ca^2+^ signaling is often involved in the onset and progression of cardiovascular diseases, such as cardiac hypertrophy, heart failure, arteriosclerosis, and hypertension. In excitable cells, such as cardiac myocytes and vascular smooth muscle cells (VSMCs), membrane depolarization, and the subsequent elevation of cytosolic Ca^2+^ concentration ([Ca^2+^]_cyt_) via voltage-dependent Ca^2+^ channels (VDCCs) cause muscle contraction, which is known as excitation–contraction coupling (E-C coupling). Elevated [Ca^2+^]_cyt_ can also activate Ca^2+^-dependent enzymes, in some cases leading to changes in gene expression patterns and contributing to long-term cellular responses. This mechanism is referred to as excitation-transcription coupling (E-T coupling), and it is involved in both the adaptive and pathological responses of the cardiovascular system to chronic stimulation. Specific intracellular regions, known as Ca^2+^ microdomains, exhibit localized increases in [Ca^2+^]_cyt_. Such localized Ca^2+^ signaling is now known to be one of the molecular mechanisms controlling the diversity of Ca^2+^ responses. These Ca^2+^ microdomains are often formed by complexes consisting of Ca^2+^ channels and downstream Ca^2+^-dependent enzymes localized by scaffolding proteins. This review outlines some of the molecular mechanisms and roles of Ca^2+^ microdomain-based E-T coupling in cardiac myocytes and VSMCs. First, we discuss the major molecular components that are essential for functional Ca^2+^ microdomains. For example, VDCC (Ca_V_1.2 channel), ryanodine receptor (RyR), Ca^2+^-dependent enzymes (Ca^2+^/CaM-dependent kinase [CaMK], calcineurin [CaN], and calpain), and scaffolding proteins (A-kinase anchoring proteins [AKAPs], caveolin, and junctophilin). Next, we discuss the roles of Ca^2+^ microdomain-based E-T coupling in physiological and pathophysiological remodeling in cardiac myocytes and vascular smooth muscle cells.

## Background

Ca^2+^ signals play a critical role in maintaining cardiovascular homeostasis, including processes such as the spontaneous heartbeat, blood pressure regulation, and adaptation to external environmental changes [1]. Cytosolic Ca^2+^ concentration ([Ca^2+^]_cyt_) is precisely regulated in a spatiotemporal manner by various ion channels, ion pumps, ion exchangers and Ca^2+^-binding proteins. Disruptions in this Ca^2+^ homeostasis mechanism are known to be involved in the onset and progression of cardiovascular diseases, including cardiac hypertrophy, heart failure, atherosclerosis, and hypertension [2, 3].

Cardiac myocytes, the dominant cell type in the heart, and vascular smooth muscle cells (VSMCs) in the media of blood vessels, are excitable cells. In response to various stimuli, their transmembrane potential depolarizes, leading to Ca^2+^ influx through voltage-dependent Ca^2+^ channels (VDCCs) and a subsequent large increase in [Ca^2+^]_cyt_ [4, 5]. This elevation in [Ca^2+^]_cyt_ triggers muscle contraction, a phenomenon known as excitation–contraction coupling (E-C coupling), which underlies fundamental cardiovascular functions such as the phasic contraction or heartbeat and blood pressure regulation [6]. Beyond its role in contraction, the increase in [Ca^2+^]_cyt_ also activates Ca^2+^-dependent enzymes, often leading to changes in gene expression patterns and contributing to a range of other long-term cellular responses. This mechanism, termed excitation-transcription coupling (E-T coupling), is involved in the adaptive responses of the cardiovascular system to chronic stimulation, as well as in pathological remodeling [2, 7].

In recent years, localized increases in [Ca^2+^]_cyt_ within confined regions of the various cardiovascular cell types, known as “Ca^2+^ microdomains”, have been implicated in the spatial localization and selectivity of Ca^2+^ signaling pathways [8]. Ca^2+^ influx through VDCCs elicits a global increase in [Ca^2+^]_cyt_ throughout most cells, triggering cellular responses, such as E-C coupling [6]. However, in addition, in defined spaces that are in proximity to the plasma membrane, sarcoplasmic reticulum (SR), and nucleus, complexes formed by Ca^2+^ channels, downstream Ca^2+^-dependent proteins, and scaffolding proteins enable localized [Ca^2+^]_cyt_ increases and signal transduction [9]. The formation of these Ca^2+^ microdomains is now known to allow for stimulus-selective cellular responses. These Ca^2+^ microdomains play an important role in the signaling selectivity and diversity of E-T coupling.

This review outlines some of the main molecular mechanisms by which Ca^2+^ microdomains enable the specificity and diversity of E-T coupling in cardiac myocytes and VSMCs. We particularly focus on (i) how Ca2⁺ microdomains are reorganized by various stimuli and disease progression, and (ii) how the Ca^2+^ microdomain-based E-T coupling contributes to physiological and pathophysiological remodeling in cardiac myocytes and VSMCs.

## Molecular components of Ca^2+^ microdomains in cardiac myocytes and vascular smooth muscle cells

### Voltage-dependent Ca^2+^ channels (VDCCs)

VDCCs are Ca^2+^ channels that allow Ca^2+^ influx in response to membrane depolarization and have been classified into distinct subfamilies: L-type, N-type, P/Q-type, R-type, and T-type channels [10]. In both cardiac myocytes and VSMCs, the L-type Ca^2+^ channel (Ca_V_1.2) is predominantly expressed [11]. Ca_V_1.2 channel is composed of the pore-forming α1 subunit and the auxiliary subunits β, α2δ, and γ [10].

Ca_V_1.2 channels form complexes with various signaling molecules (such as Ca^2+^/calmodulin-dependent protein kinase II [CaMKII], protein kinase A [PKA], and protein kinase C [PKC]) and scaffolding proteins (such as A-kinase anchoring proteins [AKAPs] and caveolin), which strongly regulate their activity [12]. In cardiac myocytes, direct phosphorylation of Ca_V_1.2 channels by PKA has been shown to increase channel conductance and cardiac contractility [13, 14]. However, recent studies have indicated that channel activity depends on PKA-dependent phosphorylation of Rad. Rad phosphorylation causes Rad to dissociate from the β subunit of Ca_V_1.2 channels, leading to enhanced Ca_V_1.2 channel activity [15]. In VSMCs, phosphorylation of the Ca_V_1.2 α1 subunit, mediated by PKC activation following angiotensin (Ang) II treatment, or PKA activation via P2Y_11_ receptors under high-glucose conditions, enhances Ca_V_1.2 channel activity [16, 17]. Furthermore, these signaling pathways promote the formation of Ca_V_1.2 channel clusters. This functionally couples the Ca^2+^ channels and amplifies Ca^2+^ influx (cooperative gating) [12]. This cooperative gating of Ca_V_1.2 channel primarily arises from interactions between adjacent channels via the C-terminal region of Ca_V_1.2 channel [18]. Moreover, under pathological conditions, such as heart failure [19], hypertension [20], and diabetes [21], a splice variant of Ca_V_1.2 channel with a lower activation threshold (exon 9*) is upregulated, contributing to Ca_V_1.2 channel hyperactivity. Such Ca_V_1.2 channel activation contributes to enhanced E-T coupling in cardiac myocytes and VSMCs.

### Ryanodine receptor (RyR)

Ryanodine receptor (RyR) is the major calcium release channel localized in the SR [22]. In cardiac myocytes and VSMCs, the major isoform of RyR is RyR2 [23]. In cardiomyocytes, RyR2 is localized to T-tubules and coupled with Ca_V_1.2, where Ca^2+^ influx via Ca_V_1.2 activates RyR2, triggering Ca^2+^ release from the SR. This process, known as calcium-induced calcium release (CICR), forms the basis of E-C coupling [5]. RyR2 function is modulated by phosphorylation via CaMKII and PKA, which increases Ca^2+^ release from the SR [24]. Enhanced RyR2 function activates the CaN/ Nuclear factor of activated T-cells (NFAT) and CaN/Myocyte enhancer factor 2 (MEF2) pathways, leading to cardiac hypertrophy [25, 26]. RyR2 knockdown suppresses CaN activation and pressure overload-induced cardiac hypertrophy [27]. In VSMCs, spontaneous Ca^2+^ sparks from RyR2 activate large-conductance Ca^2+^-activated K^+^ (BK_Ca_) channels, generating spontaneous transient outward K^+^ currents (STOCs). This leads to membrane hyperpolarization, contributing to vasodilation [28, 29]. Smooth muscle-specific RyR2 knockout abolishes Ca^2+^ sparks and enhances myogenic contraction [30]. Furthermore, the inhibition of RyR is known to suppress membrane hyperpolarization and increase cyclic AMP response element binding protein (CREB) phosphorylation [31]. These findings suggest that RyR2 may be involved in E-T coupling in both cardiac myocytes and VSMCs.

### Ca^2+^/calmodulin-dependent protein kinases (CaMKs)

CaMKs, serine/threonine kinases, are activated upon binding of the Ca^2+/^calmodulin (CaM) complex. These are known as multifunctional kinases with multiple substrate specificities [32]. The CaMK family is divided into CaMKII, which functions as a multimeric holoenzyme, and CaMKI and CaMKIV, which exist as monomers. These kinases have different mechanisms of activation [32]. In the CaMKII holoenzyme, Ca^2+^/CaM binding induces autophosphorylation between adjacent molecules, resulting in autonomous activation [33]. In contrast, CaMKI and CaMKIV require both Ca^2+^/CaM binding and phosphorylation by the upstream kinase, Ca^2+^/calmodulin-dependent protein kinase kinase (CaMKK) [34, 35]. The CaMKK family includes CaMKK1 and CaMKK2. While CaMKK1 kinase activity is Ca^2+^/CaM-dependent, CaMKK2 exhibits partial autonomous activity even in the absence of Ca^2+^/CaM due to autophosphorylation [36].

CaMK family members can strongly modulate both intracellular Ca^2+^ dynamics and gene expression via phosphorylation. CaMKII is involved in the regulation of intracellular Ca^2+^ dynamics by phosphorylating Ca_V_1.2 channels, RyRs, and phospholamban (PLN), a regulator of SR Ca^2+^-ATPase (SERCA) [37, 38]. In cardiac myocytes, CaMKII phosphorylates histone deacetylase 4 (HDAC4), promoting its export from the nucleus and leading to cardiac hypertrophy [39, 40]. Conversely, CaMKII-mediated activation of CREB is involved in cardioprotection [41]. In VSMCs, CaMKII also contributes to VSMC hypertrophy via phosphorylation of HDAC4 [42]. On the other hand, the CaMKK2/CaMKI pathway activates CREB and is involved in vascular remodeling [43, 44].

### Calcineurin (CaN)

CaN is a Ca^2+^/CaM-dependent serine/threonine phosphatase consisting of a catalytic subunit, CnA, and a regulatory subunit, CnB [45]. CaN is activated when Ca^2+^ binds directly to CnB and the Ca^2+^/CaM complex binds to CnA, causing the autoinhibitory domain (AID) to dissociate from the catalytic site [46]. CaN also has CaM-independent activation mechanisms [47]. CaN dephosphorylates NFAT, inducing NFAT nuclear translocation and transcriptional activation [48]. In cardiac myocytes, activated NFAT often being induced by CaN cooperates with other transcription factors such as MEF2 [49], GATA [50], and NF-κB [51], and is involved in physiological responses a51nd cardiac hypertrophy. In VSMCs, activation of Ca_V_1.2 channel by angiotensin II (AngII) [52] or high-glucose conditions [53] activates the CaN/NFAT pathway and is involved in hypertension.

### Calpain

Calpain is a Ca^2+^-dependent cysteine protease that regulates protein function by cleaving a portion of target proteins [54]. Calpain-1 is activated by micromolar levels of Ca^2+^ and is involved in physiological cellular functions, while calpain-2 is activated by millimolar levels of Ca^2+^. It is thought to contribute to cellular damage and worsening of pathological conditions [54]. Activation of calpain by sustained [Ca^2+^]_cyt_ elevation is involved in T-tubule disruption (as occur in progressive heart failure) via cleavage of junctophilin 2 (Jph2) [55] and activation of CaN by cleavage of the AID [47].

### A-kinase anchoring proteins (AKAPs)

A-kinase anchoring proteins (AKAPs) form complexes with Ca^2+^ channels and signaling molecules, thus contributing to localized Ca^2+^ signaling [48, 56]. At the plasma membrane, AKAP5 facilitates the efficient phosphorylation and clustering of Ca_V_1.2 channels by concentrating adenylyl cyclase (AC), PKA, and PKC in close proximity to the channels [12]. AKAP5 knockout is associated with reduced cooperative gating of Ca_V_1.2 channel [16, 57]. Furthermore, phosphorylation of Ca_V_1.2 channel, RyR2, and PLB in response to β-AR stimulation is inhibited, altering Ca^2+^ dynamics [58]. The nuclear membrane-localized muscle-specific A-kinase anchoring protein (mAKAP) enhances the efficiency of PKA-mediated phosphorylation of RyR2 in the perinuclear SR near the T-tubules and is involved in local Ca^2+^ signaling at the nuclear membrane [58]. Both AKAP5 and mAKAP also bind to CaN, contributing to the CaN/NFAT pathway in E-T coupling [48]. AKAP18δ, localized to the SR, promotes PLN phosphorylation by positioning PKA near PLN and SERCA2 [59]. AKAP18δ scaffolds CAMKII to RyRs. While the N-terminal region of AKAP18δ inhibits CAMKII, binding at the COOH-terminal region enhances CAMKII activity and promotes PLN phosphorylation, ultimately regulating Ca^2+^ dynamics in cardiac myocytes [60].

### Caveolin and junctophilin

In cardiac myocytes, a characteristic microanatomical structure called T-tubule forms the junction between the plasma membrane (sarcolemma) and the SR. In the T-tubules, Ca_V_1.2 channels expressed in the sarcolemma and RyR2 in the SR membrane are highly concentrated, often forming clusters which enables efficient CICR [5]. Jph2, which anchors the sarcolemma to the SR, and the scaffolding protein caveolin 3 (Cav3) are crucial for T-tubule formation and its functional maintenance [61]. Jph2 not only attracts and anchors RyR2s on the SR to form clusters [62] but also localizes Ca_V_1.2 channels in close proximity to RyR2 clusters, both are essential for CICR [61]. Cardiac-specific knockdown of Jph2 leads to heart failure, reduced CICR, and abnormally increased RyR2 activity [63]. Conversely, overexpression of Jph2 suppresses RyR activity [64]. Adult cardiac myocytes that express Jph2 having mutations in the Ca_V_1.2 channel interaction site, significantly alter the distribution of Ca_V_1.2 channel and the T-tubule structure [65]. Jph2 plays a crucial role in the structural and functional maintenance of T-tubules and Ca^2+^ handling [66, 67]. Cav3, in cooperation with AKAP5, forms complexes with Ca_V_1.2 channel, AC, PKA, and β-adrenergic receptors (β-ARs), contributing to β-adrenergic signaling in cardiac myocytes [68]. In pressure overload, Cav3 expression is downregulated, and VDCC current in T-tubules is decreased　[69]. Furthermore, in Cav3 knockdown, VDCC current in T-tubules is decreased [70], and the activation of Ca_V_1.2 by β2-AR stimulation is suppressed [71].

Although VSMCs lack T-tubules, an invaginated structure called caveolae, composed of lipid rafts and caveolin, is consistently found on the plasma membrane [72]. In these caveolae, G protein-coupled receptors and distinct sets of ion channels are present at high levels, and these complexes interact with the SR, and thus regulate intracellular Ca^2+^ dynamics [72]. We have reported that caveolin 1 (Cav1), an essential component of caveolae in VSMCs, and Jph2 functionally couple BK_Ca_ channels clustered in caveolae with RyR2 on the SR. This complex contributes to generation of STOCs and can stabilize vascular tone [28, 29]. On the other hand, the Ca_V_1.2 channel, the Ca^2+^-activated Cl^−^ channel (TMEM16A), and IP_3_ receptor (IP_3_R) form clusters located between caveolae and the SR. This signaling complex augments Ca^2+^ influx from Ca_V_1.2 channel mediated by G protein-coupled receptor stimulation, such as by endothelin (ET-1) [73]. In Cav1 knockout mice, vasoconstriction induced by adrenergic stimulation is attenuated, and mean blood pressure is decreased [74] and AngII-induced vascular remodeling is suppressed [75]. Furthermore, it has been reported that the number and microanatomy of caveolae in VSMCs changes with aging [76].

## Cardiac remodeling and E-T coupling

Cardiac myocytes are exposed to various stresses, such as pressure overload and maintained excessive neurohormonal stimulation. In response to these stresses, Ca^2+^ signaling can lead to and promote adaptive remodeling, including cardiac myocyte hypertrophy. However, chronic stimulation alters the structure of the T-tubules and the nucleus, as well as the composition of Ca^2+^ microdomains formed therein, causing pathological remodeling such as fibrosis, and ultimately progressing to chronic heart failure [2, 77].

### Adaptive cardiac remodeling and E-T coupling

**Fig. 1:**
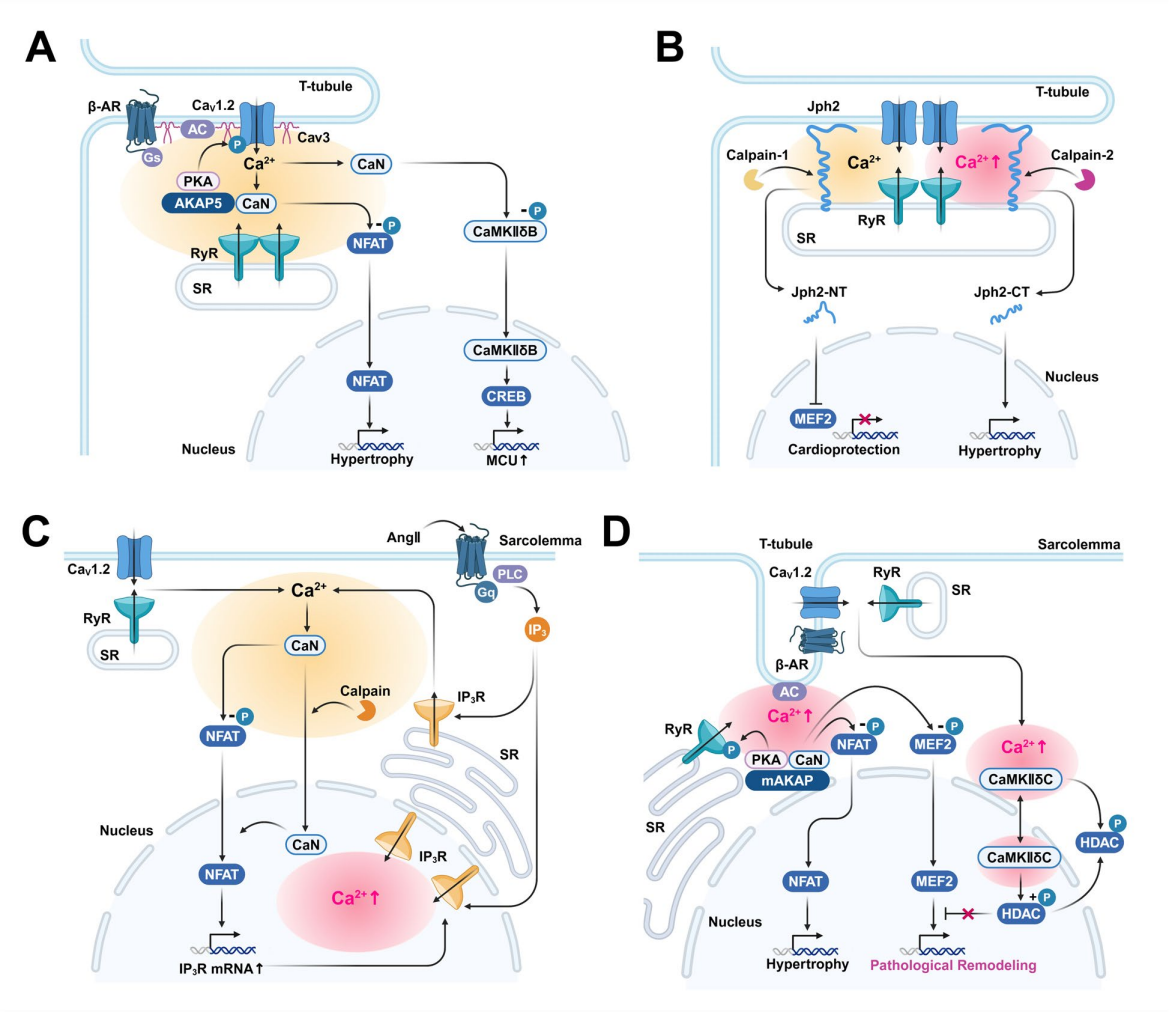
E-T Coupling in Cardiac myocytes. **A** AKAP5 localized in the T-tubules of cardiac myocytes cooperates with Cav3 to form complexes with Ca_V_1.2 channels, β-AR, AC, PKA, and CaN, leading to activation of the CaN/NFAT pathway and contributing to cardiac hypertrophy. Activation of CaMKIIδB via dephosphorylation by CaN activates CREB, inducing MCU expression and exerting cardioprotective effects. **B** Junctophilin 2 (Jph2) which tethers the T-tubules and SR is cleaved by calpain activated by sustained [Ca^2+^]_cyt_ elevation. Jph2-NT, generated by calpain-1, has a cardioprotective effect. In contrast, Jph2-CT, generated by calpain-2, is involved in cardiac hypertrophy. **C** Pressure overload and/or maintained neurohormonal stimulation increase both [Ca^2+^]_cyt_ and [Ca^2+^]_nuc_. Calpain translocates CaN to the nucleus, and activates NFAT. This results in an increase in IP_3_R expression levels, and further enhances [Ca^2+^]_nuc_ elevation. **D** Under chronic stimulation, CaMKIIδC accumulates in the perinuclear region and the nucleus, which promotes nuclear export of HDAC4, and thus activates MEF2. This can contribute to pathological remodeling. Furthermore, mAKAP on the nuclear membrane forms complexes with PKA, AC, and CaN, enhancing perinuclear Ca^2+^ signaling via RyR phosphorylation and activating the CaN/NFAT and CaN/MEF2 pathways, resulting in cardiac remodeling. AC: adenylyl cyclase, CaN: calcineurin, Cav3: caveolin 3, HDAC4: histone deacetylase 4, Jph2: junctophilin 2, Jph2-CT: C-terminal fragment of junctophilin 2, Jph2-NT: N-terminal fragment of junctophilin 2, mAKAP: muscle-specific A-kinase anchoring protein, MCU: mitochondrial Ca^2+^ uniporter, RyR: ryanodine receptor, SR: sarcoplasmic, β-AR: β-adrenergic receptor

In the initial stages of heart failure, the CaN/NFAT pathway is rapidly activated, inducing early adaptive cardiac hypertrophy [78]. AKAP5, localized in the T-tubules, acts co-operating with Cav3 to form a complex with Ca_V_1.2 channel, β-AR, AC, PKA, and CaN [68], and this can regulate the nuclear translocation of NFAT [79, 80] (Fig. 1A). In cardiac myocytes that lack AKAP5, or express AKAP5 that is deficient in the CaN-binding region, NFAT activation in response to adrenergic stimulation is inhibited [81]. Sustained pressure overload within the heart causes a decrease in AKAP5 expression, and in AKAP5 knockout mice, pathological remodeling due to such pressure overload is exacerbated [58]. Based on these findings, the CaN/NFAT pathway, mediated by AKAP5, is thought to be involved in adaptive remodeling in response to sustained pressure overload. Another example is that, sustained sympathetic activation activates CaN via β-AR signaling, inducing dephosphorylation of CaMKIIδB and its subsequent nuclear translocation. CaMKIIδB activates CREB, increasing the expression of mitochondrial calcium uniporter (MCU) and reducing cytosolic Ca^2+^ concentration, thereby suppressing myocardial damage [41] (Fig. 1A).

In cardiac remodeling, structural and functional remodeling of T-tubules is observed with disease progression, leading to disturbances in Ca^2+^ homeostasis [82]. In particular, T-tubule remodeling becomes prominent during the transition from compensatory hypertrophy to heart failure [83]. Changes in mechanical stress due to local fibrosis are implicated in T-tubule remodeling progression [84]. In T-tubule membranes exhibiting disrupted microanatomy, activation of Ca_V_1.2 channel by PKA is enhanced [85]. In addition, activation of calpain by sustained [Ca^2+^]_cyt_ elevation causes cleavage of Jph2, leading to T-tubule structural disruption and reduced efficiency of E-C coupling, promoting the progression of heart failure [86, 87]. On the other hand, cleaved Jph2 can translocate to the nucleus and functions as a transcription factor. The Jph2 N-terminal fragment (Jph2-NT), generated by calpain-1, translocates to the nucleus and inhibits MEF2, a transcription factor involved in pathological remodeling, thus acting protectively [88]. In contrast, when [Ca^2+^]_cyt_ increase dramatically and are maintained, calpain-2 is activated and generates the Jph2 C-terminal fragment (Jph2-CT), which is involved in cardiac hypertrophy [89] (Fig. 1B).

### Pathological cardiac remodeling and E-T coupling

The nucleus is a distinct intracellular compartment, separated from the cytoplasm by the nuclear membrane, and the nucleus itself functions as a Ca^2+^ microdomain [77]. Pressure overload and neurohormonal stimulation not only increase [Ca^2+^]_cyt_ but also generally cause an increase in nuclear Ca^2+^ concentration ([Ca^2+^]_nuc_) and nuclear remodeling [90]. Furthermore, these types of stimuli can increase the number of IP_3_R on the nuclear membrane, contributing to chronic [Ca^2+^]_nuc_ elevation [90, 91]. In particular, chronic AngII stimulation activates CaN via calpain and promotes nuclear translocation of CaN [47]. The increased [Ca^2+^]_nuc_ strongly activates the nuclear CaN/NFAT pathway, further increasing IP_3_R expression on the nuclear membrane [91] (Fig. 1C). Chronic pressure overload and/or prolonged stimulation by ET-1, AngII, and adrenaline cause [Ca^2+^]_nuc_ elevation, leading to long-term activation of CaMKIIδC [39, 92]. CaMKIIδC accumulates in the perinuclear region and the nucleus, and it activates MEF2 by phosphorylating HDAC4 and exporting it from the nucleus, resulting in pathological remodeling [39] (Fig. 1D).

On the other hand, RyRs in the perinuclear SR also indirectly increase [Ca^2+^]_nuc_ [90]. mAKAP, localized on the nuclear membrane, promotes phosphorylation of perinuclear RyRs by PKA via β-AR stimulation. The result of this enhanced signaling induces localized [Ca^2+^]_cyt_ elevation near the nucleus and activates the CaN/NFAT and CaN/MEF2 pathways, which are involved in pathological remodeling [25, 26] (Fig. 1D). In cardiac-specific mAKAP knockout mice, pressure overload and agonist-induced cardiac remodeling are suppressed [26].

In summary, CaN and CaMKII signals work cooperatively to promote the transition from adaptive remodeling to pathological remodeling, leading to the onset of chronic heart failure.

## E-T coupling in VSMCs

In VSMCs, Ca^2+^ influx via Ca_V_1.2 channels promotes the transcription of smooth muscle differentiation markers through Rho-associated protein kinase (ROCK), resulting in the maintenance of the differentiated state [93]. However, stimuli such as chronic blood pressure increases and/or elevation of neurohumoral factors increase Ca^2+^ influx via Ca_V_1.2 channels. The result is enhanced vascular contractility (functional remodeling) and structural changes such as thickening and stiffening of the vascular wall (structural remodeling), ultimately contributing to various diseases based on hypertension [94].

**Fig. 2:**
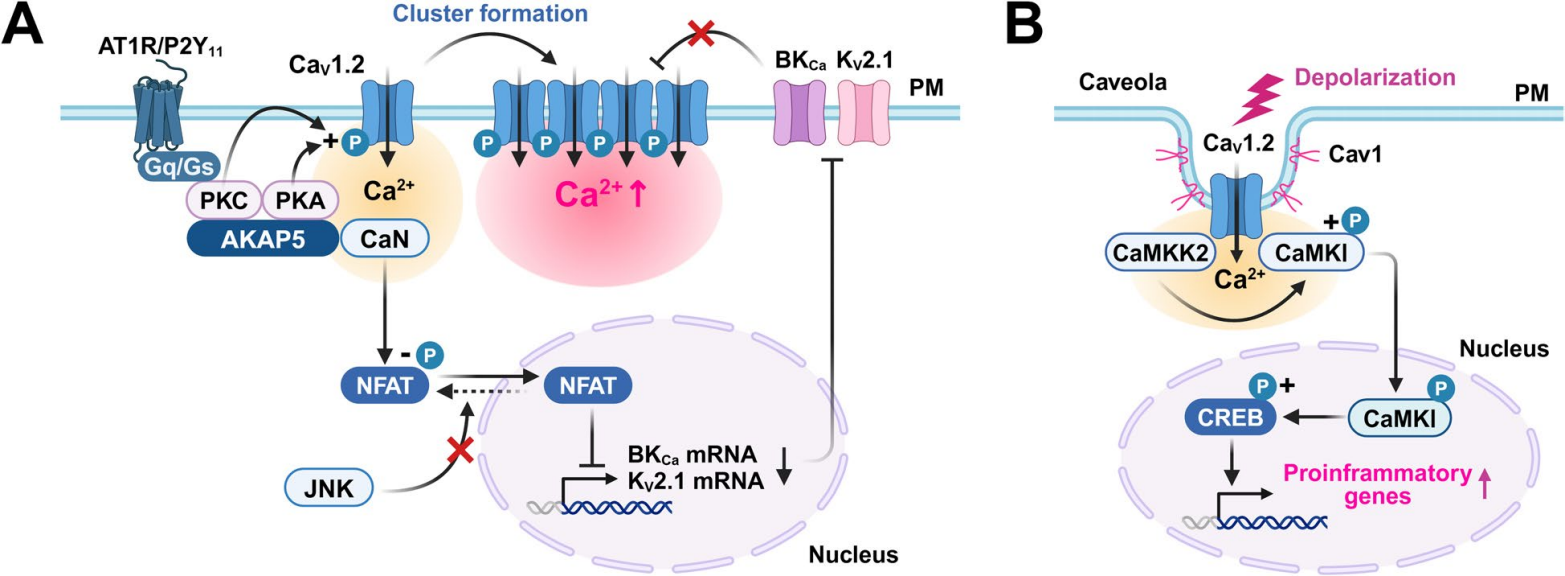
E-T coupling in vascular smooth muscle cells. **A** Phosphorylation of Ca_V_1.2 channel by AT1R/PKC or P2Y11/PKA increases Ca_V_1.2 channel clustering as well as co-operative interactions of these channels. NFAT translocates to the nucleus via dephosphorylation by CaN anchored to AKAP5. Agonist stimulation or pressure loading suppresses JNK2, stabilizing NFAT nuclear localization. Nuclear NFAT downregulates the expression of both BK_Ca_ subunits and Kv2.1, enhancing membrane depolarization. **B** Ca^2+^ influx mediated by Ca_V_1.2 channels clustered in caveolae activates CaMKK2/CaMKI in a spatially localized fashion. CaMKI, phosphorylated by CaMKK2, translocates to the nucleus and activates CREB, where it may induce genes that produce pro-inflammatory products (e.g., cytokines and chemokines). AT1R: Angiotensin II type 1 receptor, BK_Ca_: Large conductance Ca^2+^-activated K^+^ channel, CaN: calcineurin, Cav1: caveolin 1, JNK2: c-Jun N-terminal kinase 2, P2Y_11_: P2Y purinoreceptor 11, PM: plasma membrane

Both the AT1R/PKC pathway (activated by AngII) [95] and the P2Y_11_/PKA pathway (activated by high glucose) [96] phosphorylate Ser1928 of the Ca_V_1.2 α1C subunit, can promote Ca_V_1.2 channel clustering and cooperativity. AKAP5 has been reported to be required for this increase in Ca_V_1.2 channel cooperativity [12]. Activation of Ca_V_1.2 channels by AngII [52] or hyperglycemia [53] activates NFAT via CaN anchored to AKAP5, suppressing K_V_ channels function and enhancing vascular tone. In fact, AKAP5 knockout mice are hypotensive and do not develop hypertension when challenged with AngII [16] or a high-fat diet [97]. Conversely, VSMC-specific AKAP5 knock-in mice are hypertensive and exhibit thickening of the medial layer of their vessels [98]. Thus, it is thought that E-T coupling signaling pathways are selectively defined/regulated by dynamic regulation of Ca_V_1.2 channel cooperativity, and sometimes depending on the stimulus, this is mediated by membrane microdomains such as the AKAP5 complex (Fig. 2A).

Stimuli such as platelet-derived growth factor (PDGF) [99] and UTP [100] also can activate both Ca_V_1.2 channels and IP_3_R. This increases intracellular Ca^2+^ concentration and activates the CaN/NFAT system. Furthermore, pressure loading (100 mmHg) on blood vessels also activates the CaN/NFAT system in VSMCs [101]. Interestingly, Ca^2+^ influx mediated by Ca_V_1.2 channels in response to membrane depolarization alone is not sufficient to cause stable localization of NFAT in the nucleus. In fact, the aforementioned agonist stimulation and pressure loading suppress JNK2, inhibiting NFAT phosphorylation and nuclear export, thus stabilizing NFAT nuclear localization [101, 102]. Therefore, in this setting, dephosphorylation by CaN, as well as suppression of NFAT phosphorylation are both important for the induction of E-T coupling.

We have reported that Ca_V_1.2 channel, CaMKK2, and CaMKI form complexes in caveolae that are importantly involved in vascular remodeling [43, 44]. Ca^2+^ influx through Ca_V_1.2 channels in caveolae activates both CaMKK2 and CaMKI pools that are localized within caveolae. CaMKK2 further phosphorylates CaMKI [43], and this phosphorylated CaMKI then translocates to the nucleus where it induces CREB activation [44]. The phosphorylated CREB induces genes products that promote inflammation, such as inflammatory cytokines, chemokines, and leukocyte adhesion molecules. We note also that pressure loading on blood vessels can induce CREB phosphorylation, macrophage accumulation, and vascular remodeling, and that these responses are suppressed in Cav-1 knockout mice or by CaMKK2 inhibition [43] (Fig. 2B).

## Conclusion

E-T coupling plays a central role in maintaining cardiovascular homeostasis, both in adaptive responses to the external environment, and in the development of pathological conditions. However, many details of the regulatory mechanisms for E-T coupling, based on Ca^2+^ microdomains, remain incompletely understand. Several studies have reported that the expression and localization of molecules that constitute Ca^2+^ microdomains (Ca_V_1.2 channels, Ca^2+^-dependent enzymes, and scaffolding proteins) differ in normal and pathological conditions. Therefore, it is hypothesized that under pathological conditions such as heart failure and hypertension, the integrated cellular responses initiated by Ca^2+^ influx via Ca_V_1.2 channels are altered, and that this drives the transition from adaptive remodeling to pathological remodeling. Furthermore, since multiple Ca^2+^ microdomains exist within different cardiovascular cell types, crosstalk between these Ca^2+^ microdomains may take place. In particular, Ca^2+^ signaling within the nucleus has recently been reported to play an important role in E-T coupling, but the details of functional interactions between cytosolic and nuclear Ca^2+^ signals have not been extensively studied. In the future, a more complete elucidation of the regulatory mechanisms of E-T coupling, targeting Ca^2+^ microdomains, is expected to enhance our understanding of the pathophysiology of cardiovascular diseases and lead to the development of novel therapeutic strategies.

## Data Availability

Not applicable.

## Abbreviations

α-AR: α-Adrenergic receptor
AC: Adenylyl cyclase
AKAP: A-kinase anchoring protein
AngII: Angiotensin II
AT1R: Angiotensin II type 1 receptor
β-AR: β-Adrenergic receptor
BK_Ca_: Large conductance calcium-activated potassium
[Ca^2+^]_cyt_: Cytoplasmic Ca^2+^ concentration
[Ca^2+^]_nuc_: Nuclear Ca^2+^ concentration
CaMK: Ca^2+^/calmodulin-dependent protein kinase
CaMKK: Ca^2+^/calmodulin-dependent protein kinase kinase
CaN: Calcineurin
Cav: Caveolin
E-C coupling: Excitation–contraction coupling
E-T coupling: Excitation-transcription coupling
HDAC4: Histone deacetylase 4
IP_3_R: Inositol trisphosphate receptor
Jph2: Junctophilin 2
MEF2: Myocyte enhancer factor 2
NFAT: Nuclear factor of activated T-cells
P2Y_11_: P2Y purinoreceptor 11
PKA: Protein kinase A
PKC: Protein kinase C
PLB: Phospholamban
RyR: Ryanodine receptor
SERCA: Sarcoplasmic/endoplasmic reticulum Ca^2+^-ATPase
SR: Sarcoplasmic reticulum
VDCC: Voltage-dependent Ca^2+^ channel
VSMCs: Vascular smooth muscle cells
